# Biofertilizer Based on Biochar and Metal-Tolerant Plant Growth Promoting Rhizobacteria Alleviates Copper Impact on Morphophysiological Traits in *Brassica napus* L.

**DOI:** 10.3390/microorganisms10112164

**Published:** 2022-10-31

**Authors:** Adarsh Kumar, Galina Borisova, Maria Maleva, Tripti, Grigory Shiryaev, Anastasia Tugbaeva, Artem Sobenin, Irina Kiseleva

**Affiliations:** 1 Institute of Natural Sciences and Mathematics, Ural Federal University, 620002 Yekaterinburg, Russia; 2 Institute of Mining of the Ural Branch of RAS, 620075 Yekaterinburg, Russia

**Keywords:** *Bacillus altitudinis*, rapeseed, bioformulation, heavy metal, morphological parameters, lipid peroxidation, photosynthetic pigments, non-enzymatic antioxidants

## Abstract

Metal tolerant plant growth-promoting (PGP) rhizobacteria are promising for enhancing plant productivity under copper (Cu) stress. Present pot scale experiment was conducted on *Brassica napus* L. to check the efficiency of rhizobacteria isolated from the rhizosphere of *Tussilago farfara* L. growing on Cu-contaminated soils. Out of fifty Cu tolerant strains, three isolates which showed multiple PGP traits such as indole-3-acetic acid (IAA) synthesis, phosphate (PS) solubilization, siderophore and ammonia production were identified preliminarily by morphological and physiological characteristics followed by 16S rRNA gene sequencing. The best *Bacillus altitudinis* strain TF16a which showed IAA: 15.5 mg L^−1^, PS: 215 mg L^−1^, siderophore halo zone ratio of 3.0 with high ammonia production was selected to prepare a biochar-based biofertilizer (BF). Seedling test showed maximum growth of *B. napus* shoot and root in presence of 5% of BF and this concentration was selected for further experiment. The pot experiment included four treatments: control (soil), 100Cu (100 mg Cu kg^−1^ soil), 5%BF (*v/v*), and 5%BF+100Cu, which were carried out for 30 days, after which the morphological, physiological, and biochemical parameters of *B. napus* were studied. The Cu treatment caused its accumulation in shoot and root up to 16.9 and 30.4 mg kg^−1^ DW, respectively, and increased malondialdehyde (MDA) content by 20%. Application of BF with copper led to the decrease in the Cu accumulation by 20% for shoot and 28% for root while MDA content was the same as in the control. Both treatments of BF with and without Cu increased chlorophyll *a* and *b* content by 1.3 times on average as well as non-enzymatic antioxidants such as soluble phenolic compounds (1.3 times) and free proline (1.6 times). Moreover, BF + Cu led to the increase in the biomass of shoot and root by 30 and 60%, respectively, while there was no significant effect on the growth characteristics of plants after the addition of BF without Cu. The study elucidates that BF based on *B.* *altitudinis* strain TF16a and biochar can be a promising bioformulation which could increase rapeseed growth under the moderate Cu concentration in soil.

## 1. Introduction

Extensive agriculture is being practiced all around the world to fulfil the human demand from thousands of years. In recent times, numerous synthetic and chemical additives were used for cultivation of crops to enhance their productivity. Although, such additives have tremendously increased the productivity of crops, they also caused an increase in contaminants in the soil [1,2]. Heavy metals (HMs) are the widely distributed chemical pollutants which persist in the environment [3,4,5]. Copper is one of the HMs which is of great concern. Presence of Cu in the chemical fertilizers, mainly insoluble-phosphorous, as well as different pesticides increased its concentration in the cultivated crops and thus created an alarming situation [6,7,8]. According to Alengebawy et al. [7], the copper concentration in P-fertilizers varies around the world from 7 to 225 mg Cu kg^−1^ and is about 13 mg Cu kg^−1^ in European Union, whereas N-fertilizers contain 2–1450 and 1.9 mg Cu kg^−1^, respectively. Copper is an essential metal required by both plants and humans; however, the prolonged fertilizer application leads to its accumulation in agricultural soils and can cause severe health issues [9,10,11,12]. In case of plants, an increase in Cu concentration induces oxidative stress through enhanced production of various reactive oxygen species (ROS), resulting in damage of membrane lipids, enzymes, DNA, RNA, decrease in pigment and nutrient content, and plant growth [13,14]. Continuous use of these chemicals is altering the agricultural lands into unusable. Thus, there is a great need of an alternative technologies for sustainable enhanced agricultural production [2,4,7].

In past few decades, plant growth-promoting rhizobacteria (PGPR) gained significant importance in crop yield production. They are capable to improve plant nutrition by phosphate solubilizing (PS) and ammonia (AM) production; promote plant growth by producing indole-3-acetic acid (IAA); protect plants from pathogenic attacks by synthesizing hydrogen cyanide (HCN); and immobilize HMs by producing siderophores (SP) [2,3,15]. Additionally, they support soil biota by mitigating different abiotic stress factors such as HMs, drought, and salinity. However, PGPR cannot improve the physical properties of soil and thus there is a need for their amalgamation with an ecofriendly carrier to enhance the shelf-life and efficiency.

Numerous solid carrier substrates such as coal, alginate, peat, compost, perlite, talc etc., were used to support the PGPR [4,16]. Biochar is also one of such substrates which is generated by thermochemical conversion of biomass in limited oxygen supply. The processing of biomass to biochar involves drying, grinding, pyrolysis, and separation [2]. Biochar is a carbonaceous material which possess multitudinous pores which can retain water, therefore can improve moisture content (MC) when applied to the soil. Its high specific surface area, water holding capacity (WHC) along with presence of carbon, hydrogen, nitrogen, macro-, and micronutrients with several nonpolar or polar substances, which have a strong affinity to inorganic ions such as HMs ions, nitrate, and phosphate makes it the most suitable carrier substrate for amalgamation with PGPR to prepare a stable and efficient biofertilizer [2].

*Brassica napus* L. (rapeseed) is an oil yielding fast growing plant with large biomass, used as vegetable. It can sequester the majority of metals in the underground part, thus participates in production of clean above-ground biomass and yield [17].

In the present study, we hypothesize that biofertilizer (BF) prepared from wood biochar combined with metal-tolerant PGPR can improve the biometric growth parameters and alleviate the copper impact on the morphophysiological traits of *B. napus*. The specific objectives of the study include: (a) isolation, identification, and characterization of metal tolerance and plant growth-promoting (PGP) traits (IAA, PS, SP, AM, and HCN) of rhizobacteria from Cu-contaminated site, (b) determination of biochar + PGPR based BF efficiency using Petri plate assay, and (c) evaluation of the BF influence on biometric growth parameters (shoot and root length and biomass) as well as physiological and biochemical characteristics (the content of lipid peroxidation products, photosynthetic pigments, soluble phenolic compounds, and free proline) of rapeseed plant with and without of copper.

## 2. Materials and Methods

### 2.1. Sampling and Preparation

To isolate the metal-tolerant PGPR, one of the most dominant plant species *Tussilago farfara* L. growing on Cu smelter influenced site (55°29′44.8″ N; 60°14′50.5″ E) near to Karabash town (Chelyabinsk region, Russia) was selected. The rhizospheric soil was collected in sterile zip-lock bags, and transferred to the laboratory. One-third of the soil sample was stored at 4 °C for bacterial isolation and the rest were air dried, sieved, and oven dried at 70 °C for physicochemical characterization according to the methods reported by Maiti [18].

### 2.2. Isolation, Morphological Characterization, and Genetic Identification

To isolate the rhizospheric bacteria, 10 g of soil sample (on dry weight basis) was transferred in 250 mL of Erlenmeyer flask and mixed with 90 mL of phosphate buffer (pH 6.5), shaken at 180 rpm, 2 h at 28 °C. 

The soil suspension was serially diluted to 10^−1^–10^−7^ with sterile distilled water. Luria-Bertani (LB) agar plates supplemented with Cu (10 g of tryptone, 5 g of yeast extract, 10 g of NaCl, 17 g of agar, and 100 mg of Cu per L, pH 7.0 ± 0.2) were inoculated with 100 μL of serially diluted samples and incubated at 28 °C for next three days in a microbiological incubator to obtain the separate colonies. Morphologically different colonies were picked carefully and further grown on LB-media to obtain pure colonies. The isolates were preliminarily identified by their morphology (color, texture, shape, growth, height, size, pigmentation and optical property), Gram staining, and verified by Bergey’s manual [19].

Briefly, genetic identification was based on sequencing the 16S rRNA gene. It was amplified by PCR using genomic DNA as a template and bacterial primers, 27f (50-AGAGTTTGATCMTGGCTCAG-30) and 1492r (50-TACGGYTACCTTGTTACGACTT-30). The automated sequencing was performed as described by Kumar et al. [15]. The obtained sequences were tallied to nucleotide sequences in GenBank using the BLASTn program. The Mega 11 software was used for phylogenetic analysis. The 16S rRNA sequences were aligned using ClustalW. The phylogenetic tree was constructed using the Neighbor-Joining Method, genetic distances were generated using the Tamura-Nei model. The numbers at the branches are bootstrap confidence percentages from 1000 bootstrapped trees.

### 2.3. Minimum Inhibitory Concentration, PGP Attributes, Drought Tolerance and Antibiotic Sensitivity Test

The minimum inhibitory concentration (MIC) was determined using the methods reported by Kumar et al. [15]. Briefly, the isolates were subjected to increasing metal concentrations (for Cu: 250–1750 mg L^−1^; Cd: 50–750 mg L^−1^; Cr: 100–1000 mg L^−1^; Pb: 500–3500 mg L^−1^ and Ni: 250–2250 mg L^−1^), allowed to grow for 5 days at 28 °C in agar plate until their growth arrests at particular concentration. Precipitation of metals was prevented by adding EDTA (ethylenediaminetetraacetic acid). Polyethylene glycol (PEG) 6000 was used to test the level of drought tolerance of isolated strains by growing them in tryptone yeast-extract and glucose (TYEG) media for 3 days at 28 °C supplemented with PEG 6000 (5% to 25%) and compared with the control [15]. PGP attributes such as IAA and SP were estimated by the method reported by Brick et al. [20] and Schwyn and Neilands [21], respectively, whereas P-solubilization and AM production were checked as described earlier [4]. Antibiotic sensitivity test was performed according to Kumar et al. [15].

### 2.4. Carrier Characterization, Biofertilizer Preparation, and Survival Test of Strain TF16a

The biochar produced from birch wood, obtained from domestic manufacturer (Russia) was sieved through 300 mesh size followed by drying for 2 days at 70 °C, and double sterilized at 110 °C for 20 min [2]; and tested for its physicochemical properties. The pH and electrical conductivity (ES) of biochar were determined in water slurry (1:5, *w/v*). The MC and WHC were determined by standard methods [22].

For BF preparation, the strain of metal-tolerant and drought-resistant bacteria (*Bacillus altitudinis* strain TF16a) was grown overnight in 250 mL LB broth (10 g of tryptone, 5 g of yeast extract, 10 g of NaCl per L, pH 7.0 ± 0.2) for 2 days shaking at 150 rpm at 28 °C on orbital shaker-incubator (Biosan ES-20/60, Riga, Latvia). The bacterial culture was centrifuged at 5000× *g* rpm, 10 min and the pellet was washed twice with phosphate buffer (pH 6.5). The obtained pellet was diluted to 99 mL (~10^8^ cfu mL^−1^) and added with 1 mL glycerol. The cell suspension was aseptically added to 250 g of biochar, and left overnight in laminar over flow until the moisture level reached 25–35%. The prepared biofertilizer was sealed in sterile bags and stored at room temperature for use in seedling and pot scale experiments. 

The BF was tested to check the shelf-life by comparing its cfu count at the beginning and the end of the three-month time period. Five grams of BF was suspended in 45 mL sterile buffer, shaken at 160 rpm, 45 min at 28 °C, serially diluted, put on LB plates, and cfu were counted and recorded after 3 days of transfer.

### 2.5. Seedling Growth Test and Plant Development Assay

The healthy seeds of *B. napus* were soaked in sterile Millipore water for 24 h at room temperature. Four treatments: 0%BF (Millipore water, control), 2.5%BF, 5%BF, and 7.5%BF (BF:Millipore water, *w/v*) were prepared and 20 fully imbibed seeds were transferred on Petri plates fitted with circular sterile filter paper (five replicates for each treatment). The seeds were grown for 7 days in a plant growth chamber under 14:10 (day:night) photoperiod, illumination 150 ± 20 µM m^−2^ s^−1^ provided by phytolamps (ULI-P10-18W/SPFR IP40) at 23 ± 2 °C. The percentage of seed germination was calculated as the number of seeds germinated to the total number of seeds sown × 100. The length and biomass of shoot and root were recorded.

The pot experiment was carried out under controlled conditions in September 2020 and repeated in September–October 2021, and the results were averaged. Three *B. napus* plants were grown in 150 mL plastic pots (nine replicates for each treatment) and placed in growth chamber for 30 days under the conditions as described above. Four treatments: control (soil without Cu and BF), 100Cu (100 mg Cu kg^−1^ soil), 5%BF (BF:soil, *v/v*), and 5%BF+100Cu were prepared. The seeds of *B. napus* were sown in double sterilized (130 °C for 15 min) garden soil which showed pH 6.5 ± 0.2, EC 0.4 ± 0.02 dS m^−1^, and organic carbon 1.75 ± 0.2%. The content of Cu in the soil was low and did not exceed 20.5 ± 2.5 mg kg^−1^. The following growth characteristics of plants were studied: the rate of seed germination [23], length of shoot and root, dry biomass of aboveground and underground organs, and leaf area [15]. Plant organs were properly washed, dried, and used for Cu determination. Wet ashing of plant samples was carried out by digesting 100 mg sample using 70% nitric acid on a hot plate until the full dissolution was achieved. The Cu content in *B. napus* organs was determined by the atomic absorption spectrometer AA240FS (Varian Australia Pty Ltd., Mulgrave, Victoria, Australia).

### 2.6. Physiological and Biochemical Parameters

Physiological and biochemical parameters of *B. napus* leaves were determined spectrophotometrically using multimode plate reader Infinite M 200 PRO (Tecan, Grödig, Austria). The content of lipid peroxidation products was measured based on the level of malondialdehyde (MDA) in the reaction with thiobarbituric acid by the absorbtion at 532 and 600 nm [24]. Extraction of photosynthetic pigments was carried out in 80% acetone. The optical density of extracts was measured at 470, 647, and 663 nm and the content of pigments was calculated according to Lichtenthaler [25]. The amount of soluble phenolic compounds (including flavonoids) in plant leaves was determined in 80%-ethanol extracts (24-h extraction in darkness) as described earlier [26]. The total phenolic content was measured in the reaction with the Folin–Ciocalteu reagent at 725 nm. Gallic acid (Sigma-Aldrich Chemie GmbH, Taufkirchen, Germany) was used as a standard [27]. The amount of flavonoids was measured at 412 nm after reacting with 10% aluminum chloride, using rutin (Sigma-Aldrich Chemie GmbH, Taufkirchen, Germany) as a standard [28]. The content of free proline was determined after extraction in boiling water (95–100 °C). Staining was performed with a ninhydrin solution with the addition of glacial acetic acid in an equivalent ratio according to a modified method at a wavelength of 520 nm [26].

### 2.7. Statistical Analyses

Statistical processing of the results was carried out using STATISTICA 10.0 and Excel 16.0 software. After checking the normality by Shapiro–Wilk test and the homogeneity of variance by Levene’s test, the differences between the studied treatments were determined with the nonparametric Kruskal–Wallis H test and Mann–Whitney U test at *p* < 0.05. The relationship between different parameters was estimated by Spearman’s rank correlation coefficient. The figures and the tables show the arithmetic mean values (means) and their standard deviations (SD), significant differences between the treatments are indicated by different alphabetical letters. 

## 3. Results and Discussion

### 3.1. Chemical Properties and Heavy Metal Content in Rhizospheric Soil and Biochar

The rhizospheric soil of *T. farfara* was found slightly acidic in nature (5.40 ± 0.30) with low electrical conductivity (0.3 ± 0.09 dS m^−1^) and organic carbon content (0.35 ± 0.03%). The soil was found rich in nickel content (550 ± 20 mg kg^−1^) and had a high magnesium:calcium ratio (4.85 ± 0.25) which reflects its serpentine nature. Because of the continuous operation of century old Cu smelter and geogenic origin, the soil showed maximum concentration for Cu (3058 ± 102 mg kg^−1^) followed by manganese (991 ± 32 mg kg^−1^), lead (869 ± 24 mg kg^−1^), zinc (786 ± 21 mg kg^−1^), and chromium (566 ± 18 mg kg^−1^). The biochar used in experiments had circumneutral pH (6.9 ± 0.2) with EC (0.3 ± 0.02 dS m^−1^), MC (14.0 ± 0.4%), and WHC (81.5 ± 2.5%).

### 3.2. Bacterial Identification and Characterization

A total of fifty morphologically different isolates, which can tolerate 100 mg Cu L^−1^, were obtained from rhizospheric soil adhered to the roots of *T. farfara*. Out of fifty, only twelve isolates, which were able to tolerate Cu concentration above 750 mg L^−1^ and Ni above 1250 mg L^−1^, were further tested for PGP attributes. These isolates were capable to produce IAA and solubilize phosphates, whereas only five produced siderophores. Ammonia production was observed by seven isolates, whereas only one isolate demonstrated HCN production. Three isolates which showed at least four out of five PGP attributes were selected for identification. The 16S rRNA gene sequencing showed >99% similarity of these strains to the reported NCBI database *Bacillus* sp. (strain TF16a), *Arthrobacter* sp. (strain TF16b), and *Pseudomonas* sp. (strain TF16c). *Bacillus*, *Arthrobacter,* and *Pseudomonas* species were shown to play an important role in plant growth and development in metal contaminated sites and have been mostly described in the literature [2,15,29].

### 3.3. Minimum Inhibitory Concentration, PGP Attributes, and Drought Resistance of Selected Strains

The characteristics of three selected strains are presented in Table 1. High Cu tolerance was exhibited by all three strains and ranged between 750 and 1750 mg L^−1^, whereas for Ni it was 1250–2250 mg L^−1^. They were also able to tolerate a high concentration of Pb, Cr, and Cd. 

The rhizosphere of metal-tolerant plant possess different beneficial microorganisms that play a vital role in plant growth. They can produce IAA which promotes root elongation; chelate Fe and other metals by producing siderophore which help in nutrient uptake; solubilize phosphates by excretion of organic acids; and produce ammonia for the enhanced N-uptake [3,16]. For PGP traits, IAA production was found maximal for strain TF16b, whereas P-solubilization for TF16a (Table 1). The halo zone revealed by strain TF16a was maximal among three strains which evidenced its high siderophore producing ability. Ammonia was produced by both TF16a and TF16b strains, whereas none of the three strains showed positive result for hydrogen cyanide (HCN) production. 

Drought stress induced by PEG 6000 from −0.05 to −0.73 MPa showed that both TF16a and TF16b strains were capable to tolerate moderate-to-high stress up to −0.73 MPa (25% of PEG 6000). Antibiotic sensitivity test (ampicillin: 10 µg, kanamycin: 30 µg, chloramphenicol: 30 µg, penicillin: 6 µg, tetracycline: 30 µg and streptomycin: 30 µg) revealed resistance to all studied antibiotics, except for streptomycin for TF16a strain. Microorganisms living in rhizosphere of metal stressed soils are often capable to resist drought and antibiotics [3,15]. Among three strains, TF16a revealed the highest siderophore production that was important for chelation of HMs [2,4], thus it was selected for BF preparation by inoculating biochar. Shelf-life study of BF showed no significant decrease in the cfu count with only 3% decrease in MC and no significant change in pH after 90 days of storage.

**Figure 1:**
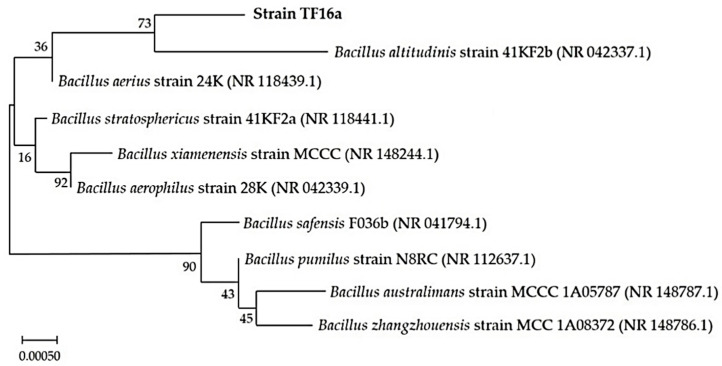
Phylogenetic tree based on partial sequences of the 16S rRNA gene of *Bacillus altitudinis* strain TF16a and identified bacteria from the NCBI database.

The high sequence similarity and phylogeny based on ClustalW indicates that strain TF16a belongs to *Bacillus altitudinis* (Figure 1). The 16S rRNA sequence was submitted into NCBI with the accession number OK103906. The phylogenetic trees showing the relationship of partially sequences 16S rRNA gene of TF16b and TF16c strains are presented in Figures S1 and S2.

### 3.4. Biometric Growth Parameters and Copper Accumulation

#### 3.4.1. Seedling Growth Test

**Figure 2:**
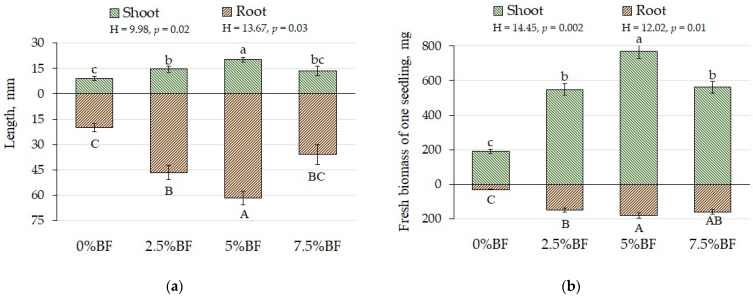
*B. napus* seedling growth parameters: (**a**) length, and (**b**) fresh biomass of shoot and root (means ± SD; *n* = 5). Different alphabetical letters indicate a significant difference at *p* ˂ 0.05.

Seedling growth test was performed in sterile Petri plates using different proportion of BF i.e., 0%, 2.5%, 5%, and 7.5%. Application of BF significantly increased the percentage of seed germination and was found maximal for 5%BF (95%) followed by 2.5%BF (90%) = 7.5%BF (90%) > control (70%) after 7 days of seedling growth. In our previous experiment on *B. napus,* different concentrations of woody biochar (from 2.5% to 10%) without PGPR did not affect the seed germination. However, application of biochar at all studied concentrations reduced the root length, but did not considerably change the shoot length and seedling biomass [30]. In the present study the maximal seedling shoot and root length (Figure 2a) and fresh biomass (Figure 2b) were found with 5%BF which could be due to the presence of PGPR strain TF16a together with biochar. We suppose that drought resistance of TF16a provided chance to plants to grow efficiently even in limited water conditions. Water deficit could be further compensated by biochar through retaining the moisture in its porous structure for a longer time [4].

#### 3.4.2. Pot Scale Experiment

The application of biofertilizers can affect the morphological parameters of plants [4,15]. Seedling growth test showed maximum growth with 5%BF application; thus, it was selected further for pot scale experiment (Figure 2). In our study, the shoot length of *B. napus* did not change at all treatments (Table 2). At the same time, the root length increased 1.5-fold with application of BF both single and combined with Cu (Table 2). It was also noted [15,31] that the combined use of BF with Cu, Zn, and/or Cd could increase the length of plant shoot and root. 

Earlier it was reported that application of 5% biochar increased shoot length of *B. napus* [30] and *Phacelia tanacetifolia* [32], but did not affect (in the case of phacelia) or decrease (rapeseed) root length. We suppose that in the present study the opposite effect of BF based on biochar and PGP-active rhizobacteria was associated with the ability of PGPR to synthesize IAA, and thus stimulate *B. napus* root growth.

**Figure 3:**
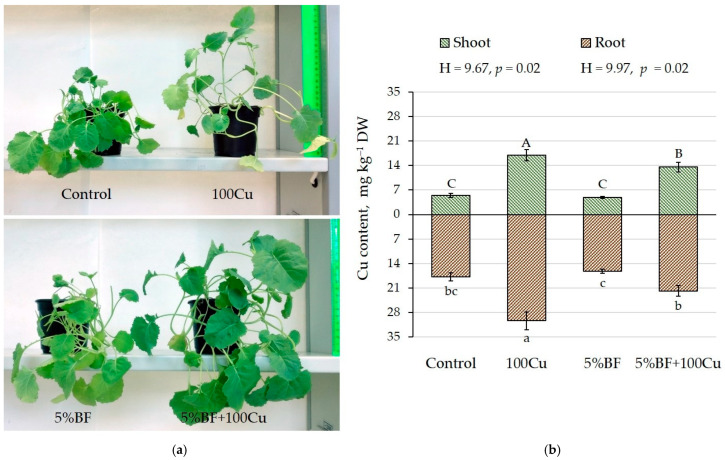
(**a**) *B. napus* plants after 30-day pot experiment, (**b**) copper accumulation in shoot and root after addition of Cu and BF (means ± SD; *n* = 9). Different alphabetical letters indicate a significant difference at *p* ˂ 0.05.

Application of 100 mg kg^−1^ of Cu led to the significant increase in the root length which suggests limited bioavailability of Cu in the soil and its transfer into the roots. Although there was no difference in shoot length, a great significant difference was observed in fresh and dry biomass of shoot and root after application of 5%BF+100Cu compared to all three treatments including control (Table 2, Figure 3a). When BF was added together with Cu, the dry weight of the shoot and root increased by 30 and 60%, respectively, and leaf area was greater than in any other treatment (Table 2). The variability of morphological parameters was not high, the coefficient of variation did not exceed 14%. 

Glick [32] and Tripti et al. [4] reported that application of metal and drought tolerant PGPR plays a vital role in shoot and root growth by alleviating the environmental abiotic stress. It can be assumed that since copper is an essential element, in the conditions of low Cu supply the application of BF could provide stimulating effect on plant growth. When copper was added separately, its concentration was too high, while BF could reduce its toxic effect due to exudates secreted by rhizobacteria [9]. 

In the present study, the addition of copper to the soil increased its accumulation in the shoot by 3.1 times and root by 1.7 times compared with the control (Figure 3b). At the same time, when copper was added together with 5%BF, its accumulation was lower by 1.3 times compared with single Cu treatment. The decrease in copper accumulation when combined with BF can be explained by metal sorption by bacteria and biochar as previously was reported [9,13,33]. Copper was mainly accumulated in the underground part of plants, while its concentration in the shoot was much lower [13,34]. Aust et al. [35] reported that effect of PGPR on the accumulation of metals in *B. napus* depended on a particular bacterial strain and bacterial genus due to different mechanisms.

### 3.5. Plant Physiological and Biochemical Parameters

**Figure 4:**
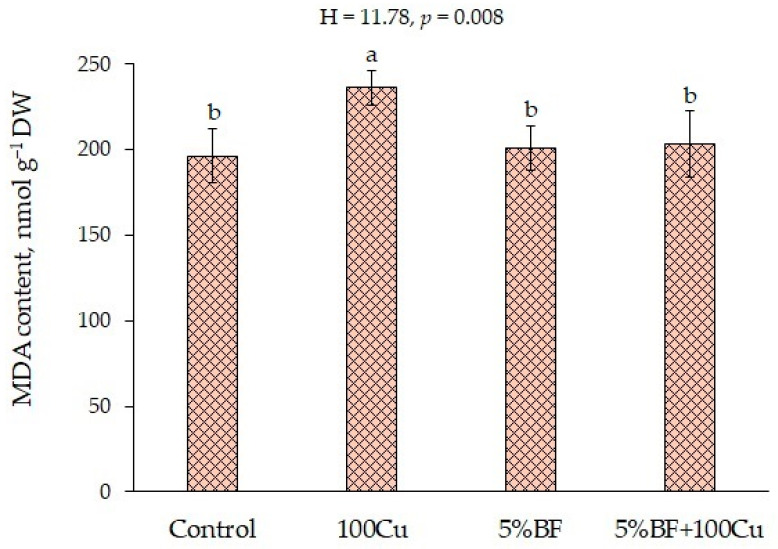
Lipid peroxidation product (MDA) content in the leaves of *B. napus* grown in soil with the addition of Cu and BF (means ± SD; *n* = 9). Different alphabetical letters indicate significant difference at *p* ˂ 0.05.

It is known that an excess of HMs can cause oxidative stress in plants due to the generation of ROS [13,14]. Since copper is a redox active metal, it is involved in the direct generation of ROS, such as superoxide radical, hydrogen peroxide, and hydroxyl radical [36]. These active molecules are involved in free radical chain reactions with membrane lipids and proteins [37]. The action of 100Cu led to a significant increase in the content of MDA in the leaves of *B. napus* by 20% (Figure 4). At the same time, after the application of 5%BF single and combined with Cu the content of MDA did not differ from the control. Similar results were observed in Cu-enriched pot scale experiment where the PGPR decreased the MDA content along with Cu accumulation in shoot and root [13]. It is possible that the use of BF contributed to maintaining the stability of membrane structures either due to partial absorption of the copper ions by biochar and/or by chelating ability of PGPR [13,34], or to the activation of the antioxidant defense system [14].

**Figure 5:**
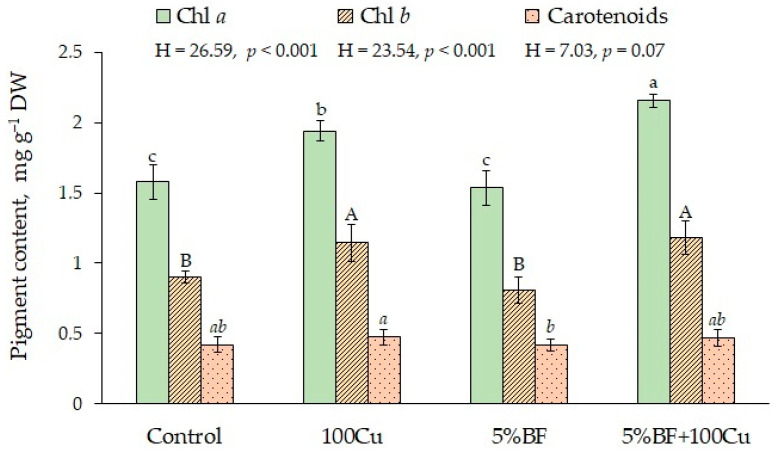
The content of photosynthetic pigments in the leaves of *B. napus* grown in the soil with the addition of Cu and BF (means ± SD; *n* = 9). Different alphabetical letters indicate significant difference at *p* ˂ 0.05.

The pigment complex is the key characteristic of photosynthetic apparatus in plants under stress. When copper was applied with and without 5%BF, the content of Chl *a* increased 1.3 times; the similar trend was found for Chl *b* (Figure 5). High positive correlation was found between Cu concentration in *B. napus* shoot and chlorophyll content in leaves (r_s_ = 0.69 for Chl *a* and 0.81 for Chl *b*, at *p* < 0.05) (Table S1). In case of separate application of BF the content of photosynthetic pigments did not change. Copper is an important essential element and plays a significant role in plant physiology, which well explains the rise of chlorophylls content when applied [9,38]. In case of carotenoids, there were no substantial differences in their content. 

To protect against ROS, plants use both antioxidant defense enzymes and non-enzymatic antioxidants such as phenolic compounds, proline, ascorbic acid, glutathione, tocopherol, etc. Under abiotic stress conditions, non-enzymatic antioxidants directly quench ROS due to the presence of certain functional groups [14]. 

**Figure 6:**
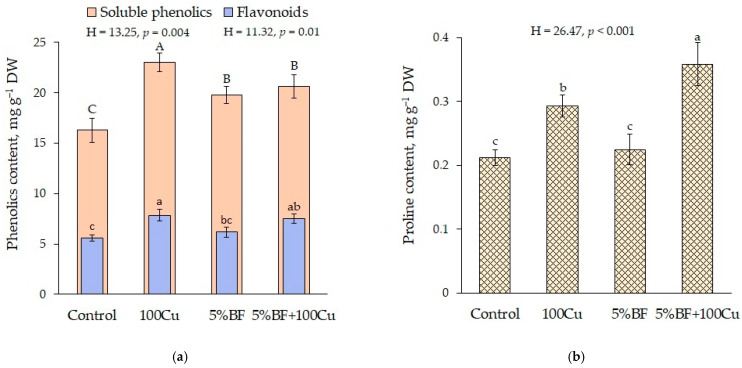
The content of (**a**) phenolic compounds and (**b**) free proline in the leaves of *B. napus* grown in the soil with the addition of Cu and BF (means ± SD; *n* = 9). Different alphabetical letters indicate significant difference at *p* ˂ 0.05.

Phenolic compounds are an important group of non-enzymatic antioxidants. Their antioxidant effect is realized in several ways: neutralization of ROS (superoxide anion and hydroxyl radicals), chelation of HMs using hydroxyl and carboxyl functional groups, as well as nucleophilic aromatic rings [39,40]. When 5%BF was added with and without Cu, the content of soluble phenolics in the leaves of *B. napus* increased on average by 25%, while in copper-treated soil without BF increased to the greatest extent by 40% (Figure 6a). This confirms their important role in the antioxidant defense of plants. Furthermore, the 5%BF+100Cu application modulated the antioxidant capability in plant which was also reported by other authors in the presence of PGPR [13]. The amount of flavonoids changed similarly to the total content of soluble phenolics: the higher amounts were found in the plants grown in Cu-treated soil (with or without BF). The proportion of flavonoids in the total content of soluble phenolics varied from 31 to 36% (Figure 6a).

One of the most important non-enzymatic antioxidants involved in the protection of plant cells from ROS is free proline. Accumulation of proline in plant cells is considered to be a non-specific defense reaction to various stress factors, including HMs [41,42]. In case of 5%BF+100Cu treatment, the content of proline significantly increased (1.7 times relative to the control) (Figure 6b). With separate addition of copper, its content also has risen by 1.4 times. It is known that PGPR can increase the accumulation of proline in cells under stress conditions and thereby increase plant resistance [43]. However, in our experiment, the increase of proline content was observed only in the presence of Cu (single and, especially, combined with BF).

Spearman’s rank correlation analysis revealed high positive correlation between Cu concentration in shoot of *B. napus* and total soluble phenolics (r_s_ = 0.77 at *p* < 0.05, Table S1), including flavonoids (r_s_ = 0.71), and free proline (r_s_ = 0.67) in leaves, which indicates a significant role of these non-enzymatic antioxidants in plant protective reactions. 

## 4. Conclusions

*Bacillus altitudinis* strain TF16a isolated from the rhizospheric soil of copper smelter influenced site showed a high tolerance to Cu and was able to exhibit multiple plant growth-promoting attributes (indole-3-acetic acid, siderophore, ammonia production, and phosphate solubilization). Application of 5% biofertilizer (BF) based on *B. altitudinis* strain TF16a and wood biochar increased the growth of *Brassica napus* in seedling growth test. Pot scale experiment with application of 5%BF, with and without Cu (100 mg kg^−1^) after 30 days of *B. napus* vegetation demonstrated that Cu treatment caused accumulation of this metal in shoot and root and increased the content of malondialdehyde (MDA) in leaves as a marker of stress. Application of BF with Cu led to the decrease in the Cu accumulation in comparison with single copper addition in *B. napus* shoot and root, while MDA content remained the same as in the control. Moreover, combined treatment enhanced the growth parameters: shoot and root length and biomass, as compared to other studied treatments. The results evidenced that application of 5%BF+100Cu not only improved the growth of the rapeseed plants but also alleviated the Cu impact by synthesizing non-enzymatic antioxidants (soluble phenolic compounds and free proline) that was confirmed by high positive correlation with Cu content in *B. napus* shoot. The study suggests that BF based on *B. altitudinis* strain TF16a and biochar can be a promising bioformulation which could increase rapeseed growth under moderate Cu concentration in soil.

## Figures and Tables

**Table 1:** Minimum inhibitory concentration (MIC), plant growth-promoting (PGP) attributes, and drought tolerance of three isolated bacterial strains.

Strain	*Bacillus* sp. TF16a	*Arthrobacter* sp. TF16b	*Pseudomonas* sp. TF16c
*MIC*, *mg L^−1^*			
Copper	1000	1750	750
Lead	3500	6000	2500
Chromium	2000	2000	1000
Cadmium	750	1000	500
Nickel	2250	2250	1250
*PGP attributes*			
Indole-3-acetic acid production, mg L^−1^	^1^ 15.48 ± 0.56 a	20.81 ± 2.85 a	4.84 ± 0.45 b
Solubilized phosphate, mg L^−1^	215.26 ± 9.45 a	49.21 ± 4.50 c	130.00 ± 6.55 b
Siderophore production *	3.00	1.75	1.71
Ammonia production	+++	+++	-
Hydrogen cyanide production	-	-	-
*Drought tolerance*, *% (MPa)*			
PEG6000 tolerance	25% (−0.75)	25% (−0.75)	20% (−0.49)

^1^ Data is presented as means ± SD (n = 4). Different alphabetical letters indicate a significant difference at *p* ˂ 0.05. * Ratio halo zone diameter to colony diameter; +++ high, - negative.

**Table 2:** Morphological parameters of *B. napus* grown in soil with the addition of Cu and BF.

Parameter	Control	100Cu	5%BF	5%BF+100Cu
Shoot length, cm	^1^ 11.63 ± 0.74 a 6.38	13.46 ± 0.93 a 6.92	10.76 ± 0.50 a 4.67	10.38 ± 0.53 a 5.09
Root length, cm	6.07 ± 0.34 c 5.60	11.33 ± 0.93 a 8.16	9.25 ± 0.51 b 5.53	9.42 ± 0.42 b 3.40
Shoot FW, mg	350.25 ± 44.50 b 12.70	338.38 ± 47.59 b 14.06	298.50 ± 37.81 b 12.67	423.25 ± 20.67 a 13.82
Root FW, mg	20.87 ± 1.52 c 7.25	25.01 ± 2.10 b 8.15	26.16 ± 0.98 b 3.74	35.32 ± 2.29 a 6.47
Shoot DW, mg	58.01 ± 6.11 b 10.54	57.91 ± 7.29 b 12.56	50.825 ± 6.09 b 11.97	75.96 ± 2.21 a 2.90
Root DW, mg	3.63 ± 0.29 b 8.07	3.67 ± 0.35 b 9.62	3.69 ± 0.14 b 3.86	5.83 ± 0.37 a 6.29
Leaf area, cm^2^	4.23 ± 0.38 bc 8.85	4.64 ± 0.31 b 6.67	3.98 ±0.14 c 3.47	5.70 ± 0.31 a 5.43

^1^ Data are presented in the numerator as means ± SD (*n* = 9); in the denominator as a coefficient of variation (CV), %. Different alphabetical letters indicate a significant difference at *p* ˂ 0.05. FW: fresh weight; DW: dry weight; BF: biofertilizer.

## Data Availability

Data is contained within the article.

## Supplementary Materials

Supporting information can be downloaded at: https://www.mdpi.com/article/10.3390/microorganisms10112164/s1. Table S1: Spearman’s rank correlation between Cu concentration in shoot of *B. napus* and studied physiological and biochemical parameters. Figure S1: Phylogenetic tree based on partial sequences of the 16S rRNA gene of *Arthrobacter* sp. strain TF16b with other related sequences and identified bacteria from the NCBI database. Figure S2: Phylogenetic tree based on partial sequences of the 16S rRNA gene of *Pseudomonas* sp. strain TF16c with other related sequences and identified bacteria from the NCBI database.
