# Supplementary material for: Biofertilizer Based on Biochar and Metal-Tolerant Plant Growth Promoting Rhizobacteria Alleviates Copper Impact on Morphophysiological Traits in Brassica napus L
Source: Microorganisms. 2022 Oct 31;10(11):2164. doi: 10.3390/microorganisms10112164 (PMC9695043; doi:10.3390/microorganisms10112164)
Supplement: Supplementary file 1 [file microorganisms-10-02164-s001.zip › microorganisms-1975254-supplementary.pdf]

Table S1. Spearman's rank correlation between Cu concentration in shoot of *B. napus* and studied physiological and biochemical parameters.

Parameter	Cu content in shoot
MDA	0.455 *
Chl *a*	0.688 *
Chl *b*	0.813 *
Carotenoids	0.542 *
Total soluble phenols	0.768 *
Flavonoids	0.706 *
Proline	0.669 *

Asterisks (*) indicate significant correlations at $p < 0.05$ (n = 36).

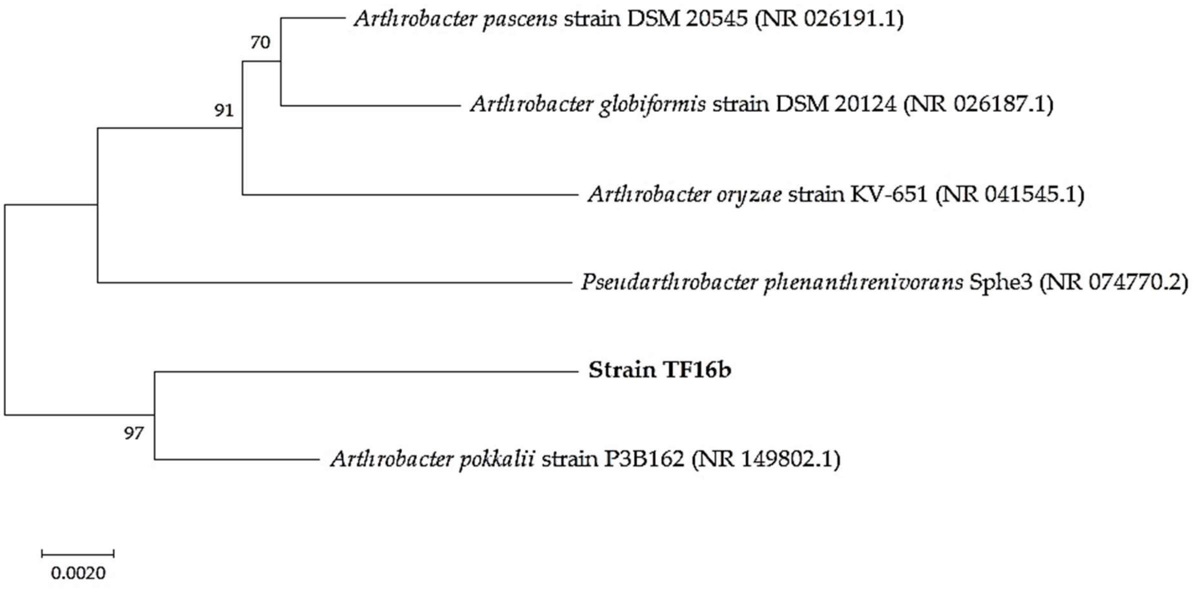

Figure S1. Phylogenetic tree based on partial sequences of the 16S rRNA gene of *Arthrobacter* sp. strain TF16b with other related sequences and identified bacteria from the NCBI database.

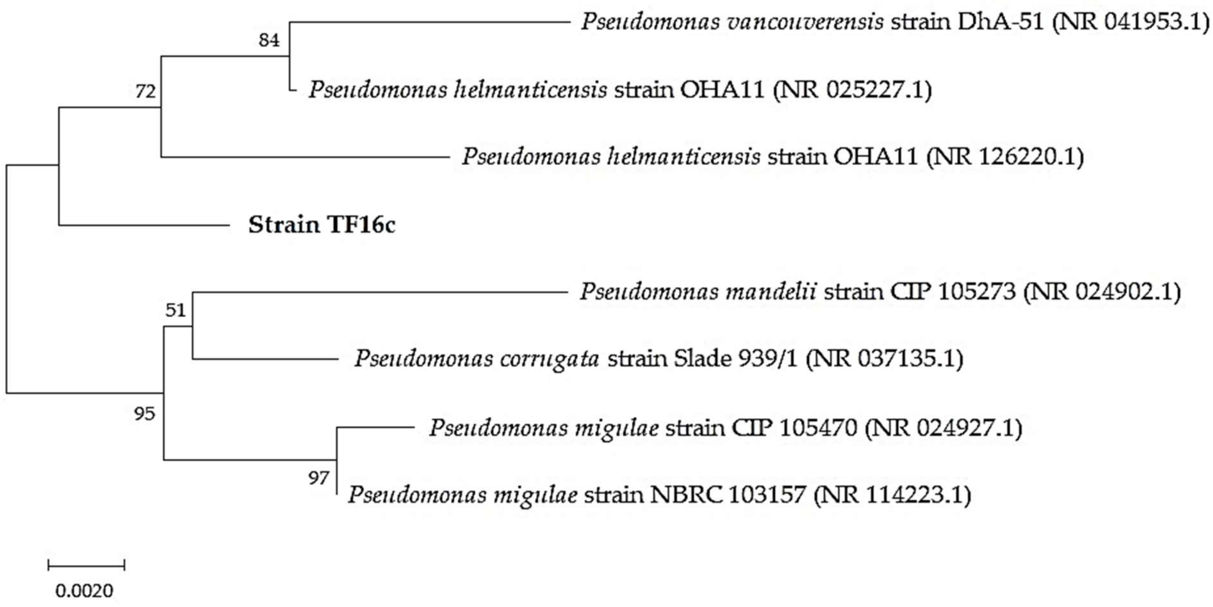

Figure S2. Phylogenetic tree based on partial sequences of the 16S rRNA gene of *Pseudomonas* sp. strain TF16c with other related sequences and identified bacteria from the NCBI database.
